# Natural and Anthropogenic Disturbances Modulate Plant Diversity in Coastal Dunes of the Northern Colombian Caribbean

**DOI:** 10.3390/plants15050671

**Published:** 2026-02-24

**Authors:** Liliana Ojeda-Manjarrés, M. Luisa Martínez, Carmelo Maximiliano-Cordova, Alejandro R. Villa, María A. Negritto, Octavio Pérez-Maqueo

**Affiliations:** 1 Facultad de Ciencias Básicas, Universidad del Magdalena, Santa Marta 470004, Colombia; 2 Instituto de Ecología A.C. (INECOL), Xalapa 91073, Veracruz, Mexico; carmelo.maximiliano@inecol.mx (C.M.-C.); alexvilla5920@gmail.com (A.R.V.); octavio.maqueo@inecol.mx (O.P.-M.)

**Keywords:** coastal dunes, Colombian Caribbean, anthropogenic impact, conservation, plant biodiversity, environmental factors

## Abstract

The conservation status of the Colombian Caribbean dune system was assessed considering the influence of natural and anthropogenic factors. The study took place in five locations with a gradient of human disturbance. In total, 198 plots and 22 transects were established, three transects in Gairaca and Costa Verde; four in Lipe, and six in Mendihuaca and Salguero. Environmental variables such as dune height, slope, sediment physical–chemical attributes, and anthropogenic impact were assessed in each site, while species composition, frequency, and plant cover were determined for each plot. The results show a correlation between natural and anthropogenic factors and the composition and structure of plant communities growing on the beach and coastal dunes. Human disturbances (urbanized areas, construction, burning, debris, trampling, logging, tourism, groins, sewage, roads, garbage, and sediment extraction) were particularly relevant. Plant cover and species diversity were inversely related to human impact and disturbance. Furthermore, community structure varied among sites: trees and vines were more frequent in the preserved locations, while shrubs and parasitic plants were more abundant in the disturbed sites. Management alternatives should consider the environmental factors (natural and anthropogenic) affecting vegetation to improve the conservation of plant diversity on coastal dunes along the Colombian Caribbean coast.

## 1. Introduction

Coastal dune systems are dynamic barriers formed by the accumulation of terrigenous and marine sand transported by the wind [1]. These systems provide ecosystem services by controlling floods and storm surges, modulating the effects of extreme events on human systems, acting as rainwater filtration zones, providing essential habitat for plants and invertebrates, and serving as feeding and nesting sites for birds and turtles. They also provide very relevant recreational services [1,2,3,4]. Coastal dunes serve as regulators of natural hazards [1,5,6] and stabilizers of the coastline, becoming the most relevant sedimentation environments on the planet [7,8,9].

Highly specialized (such as psammophilous and halophytic plants), rare, and endangered species abound in these environments [10]. Vegetation plays an important role in the dynamics of coastal dunes. Pioneer plants (mostly psammophilous and halophytic) promote dune formation by acting as obstacles that slow or stop the movement of wind-driven sediments [11]. In later stages of ecological succession (mainly on foredunes or transgressive dune fields), plant communities stabilize dune systems, retain sand, and help mitigate erosion by wind, rain, and waves by decreasing sand mobility. In addition to its protective and stabilizing role on the coast [9,12], vegetation increases the landscape value and tourist appeal of beaches [13].

Vegetation modulates the geomorphology of coastal dune ecosystems by decreasing the movement of sand and creating different dune types [14,15]. Although numerical models capture some biomorphogenic complexities, such as root reinforcement that reduces erosion [16] and the integration of airflow with vegetation dynamics [17], direct implementations of biogeomorphological interactions remain limited [16]. Ecological–morphological couplings over long temporal scales remain largely unexplored [18,19], resulting in significant gaps in the ability to predict dune system evolution under changes in sediment supply and vegetation cover [17].

The dynamics of coastal dunes and their plant communities are commonly affected and shaped by both natural and human-induced disturbances. The proximity to the ocean determines an inland gradient that affects the distribution and composition of the flora [20,21,22]. Some environmental factors that affect the ocean–inland gradient plant distribution include salt spray and soil salinity [23], soil moisture, soil texture, organic matter content, radiation and temperatures [24], sand movement and freshwater flooding [22], terrain inclination [25], and nutrient contents [22,26]. Climatic factors, such as strong winds [24], tropical cyclones, and winter storms, alter the topography and plant communities, which contribute to the natural dynamics of the coasts [27].

Anthropogenic factors also have an increasing impact on the vegetation of the beach and coastal dunes [28]. Urban and tourist development have modified dune systems, reducing their extension and vegetation cover [29,30,31,32,33]. Waste disposal has turned these ecosystems into sinks of anthropogenic waste [34], altering the sedimentation mechanisms and structure of the dunes [35]. Other factors, such as mining, invasive species, and vehicular traffic, have negatively impacted the topography and food webs of these ecosystems [36].

Recent international studies show the influence of natural and anthropogenic factors and their interaction in explaining the variation in vegetation cover and floristic diversity in coastal dunes [28,37,38,39,40,41,42]. However, the precise degree to which these pressures, individually or in interaction, modulate the floristic diversity of the coastal dune system is still unknown. The conservation level of the beach and coastal dune vegetation can be site- or region-specific, given the local particularity of the stressing agents and their intensity. In particular, the few studies [14,43] focused on the sandy coastal systems of the northern Colombian Caribbean reveal intense natural and human-induced disturbances [36,44]. However, the floristic baseline of the region is unknown, and the natural and anthropogenic disturbance agents influencing the conservation status of beaches and dunes have not yet been identified. Such knowledge is necessary to plan appropriate strategies for the conservation and restoration of these ecosystems.

Accordingly, five study sites were selected to represent a contrasting gradient of environmental conditions and varying types and intensities of anthropogenic disturbance within coastal dune systems of the Northern Colombian Caribbean. These sites range from well-preserved beaches with minimal human intervention to areas heavily affected by urbanization, tourism, logging, and burning. In addition, key environmental variables—including dune geomorphology, substrate stability, vegetation cover, dune height and slope, and soil salinity—were considered, thereby enabling a comprehensive assessment of the combined effects of anthropogenic pressures and natural factors on floristic diversity.

Coastal dune systems of the Northern Colombian Caribbean are characterized by low dune height (Hmax < 10 m), which confers high dynamism and low geomorphological stability [45,46]; consequently, they respond rapidly to both natural and anthropogenic disturbances, frequently experiencing abrupt morphological changes [5,47]. From this perspective, in poorly studied regions such as Colombia, detailed knowledge of floristic composition is essential to support management and restoration strategies for coastal dunes [48]. Accordingly, the study of these systems becomes fundamental to understand their resilience and to assess their conservation status in highly pressured coastal environments [15,42,49,50,51,52].

In this framework, the following research questions were formulated:

(i) How does floristic diversity vary among coastal dune systems of the northern Colombian Caribbean under different levels of natural and anthropogenic disturbance?
(ii) Which natural and anthropogenic factors best explain the observed differences in dune floristic diversity?
(iii) To what extent do the observed patterns of floristic diversity and associated natural and anthropogenic factors allow inference of the conservation status of dune systems and guide region-specific management and restoration strategies?

Based on the above, the study has the following objectives: (1) to determine the impact of natural and anthropogenic factors that may be affecting floristic diversity in coastal dune systems and (2) to evaluate the conservation status among coastal dune systems in the northern Colombian Caribbean. The study explores the relationship between plant species composition and structure with environmental variables and anthropogenic impacts. Management alternatives should consider the environmental factors (natural and anthropogenic) affecting vegetation to improve the conservation of the plant diversity of the coastal dunes along the Colombian Caribbean coast.

## 2. Results

We found differences between the study sites regarding natural and human-induced agents, which, in turn, affected plant composition and community structure.

### 2.1. Environmental Heterogeneity: Natural Factors

**Figure 1:**
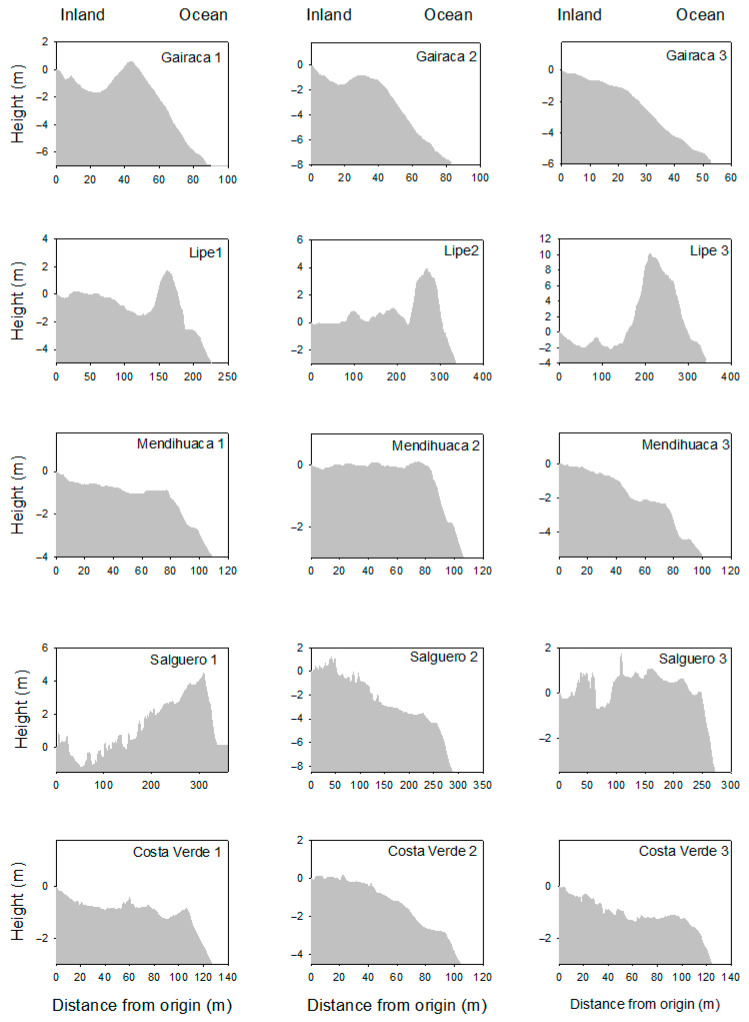
Dune profiles along three transects placed on five beaches and coastal dunes on the coast of the Colombian Caribbean. The origin represents the point (inland) where the transects for vegetation sampling started and continued towards the ocean. Negative values indicate lower heights compared with the origin, usually the highest level of the dune. These areas are exposed to increasing anthropogenic disturbances, with Gairaca being the least disturbed and Costa Verde the most.

Differences in the natural environmental factors were identified among the five sites. The most preserved sites (Gairaca and Lipe) stood out for having the highest dune heights (Figure 1), whereas the most disturbed beaches showed steeper slopes, especially near the ocean (see, for instance, Salguero and Costa Verde).

**Figure 2:**
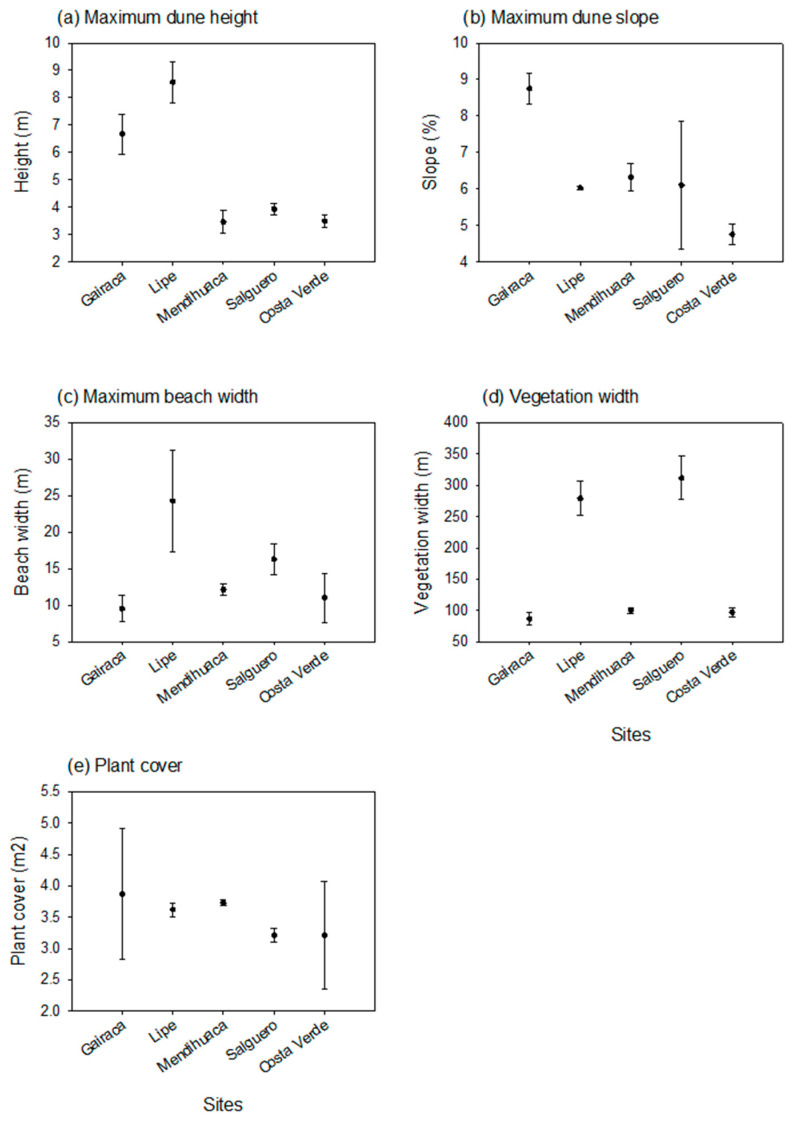
Environmental variables in five beach–coastal dune systems along the Colombian Caribbean coast. (**a**) Mean maximum dune height; (**b**) Mean maximum dune slope; (**c**) Mean beach width; (**d**) Mean vegetation width sampled, and (**e**) Mean plant cover per plot (m^2^).

The mean maximum dune slope was measured in Gairaca, while the maximum mean plant cover per plot was observed in Lipe, the second-most-preserved site. In turn, beach width was noticeably larger in Lipe than at the other sites. The width of sampled vegetation was very broad in Lipe and Salguero, whereas plant cover was highly variable in Gairaca; however, mean values were similar across sites (Figure 2).

### 2.2. Environmental Heterogeneity: Anthropogenic Factors

**Figure 3:**
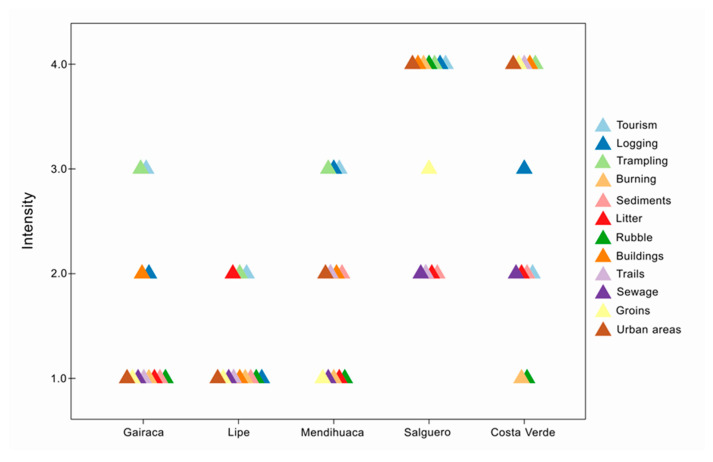
Qualitative intensity of anthropogenic disturbances in five beach–coastal dune systems along the Colombian Caribbean coast.

The analysis of 12 anthropogenic factors determined the intensity of disturbance at the five beaches. All exhibit a gradient of disturbance intensity, from low (2) to intermediate (3) and high (4). According to this analysis, Lipe and Gairaca were relatively well-preserved beaches with lower levels of anthropogenic activity. In turn, Salguero and Costa Verde are highly exposed to more frequent, more intense human disturbances (Figure 3). Urbanization, construction, and trampling were the most significant disturbance factors affecting the beaches. Mendihuaca showed intermediate conservation values because the high coverage values are associated with the presence of agriculturally important or introduced species.

The two beaches with the highest disturbance intensity were undoubtedly Salguero and Costa Verde. At Costa Verde, there was high anthropogenic disturbance in five variables: construction, trampling, groins, and roads, and intermediate disturbance in logging, with low disturbance in sewage, roads, and sediments. In turn, Salguero had the highest anthropogenic disturbance; of the 12 variables considered, seven are in the high category (urbanized areas, construction, burning, debris, trampling, logging, and tourism), 1 in the intermediate category (jetties), and 4 in the low category, in sewage, roads, garbage, and sediment extraction (Figure 3).

### 2.3. Floristic Composition

A total of 133 species, included in 113 genera and 55 families, were identified in our study sites, the northern Colombian Caribbean coast (see Appendix A). Among the most frequently found species are: *Alternanthera flavescens*, *Neltuma juliflora*, and *Pithecellobium dulce* in Lipe, Gairaca, Salguero, and Costa Verde, while creepers such as *Ipomoea pes-caprae* and *Canavalia rosea* were present in Mendihuaca, Gairaca, and Costa Verde. It is worth noting that 47 species were recorded on a single beach. Introduced species such as *Calotropis procera* and *Tribulus cistoides* were found on the beaches of Lipe, Salguero, and Costa Verde. *Hymenocallis littoralis* (Amaryllidaceae), a rare species with a limited distribution, was recorded in Mendihuaca (see Appendix A).

### 2.4. Diversity Responses to Changes in Environmental and Anthropogenic Factors

A completeness analysis was performed based on incidence, yielding ≥ 93% for Lipe, ≥89% for Gairaca, ≥93% for Mendihuaca, ≥90% for Salguero, and ≥96% for Costa Verde. A total of 133 species richness scores were recorded across the five sites: 47 at Gairaca, 44 at Mendihuaca, 34 at Lipe, 31 at Costa Verde, and 29 at Salguero.

**Figure 4:**
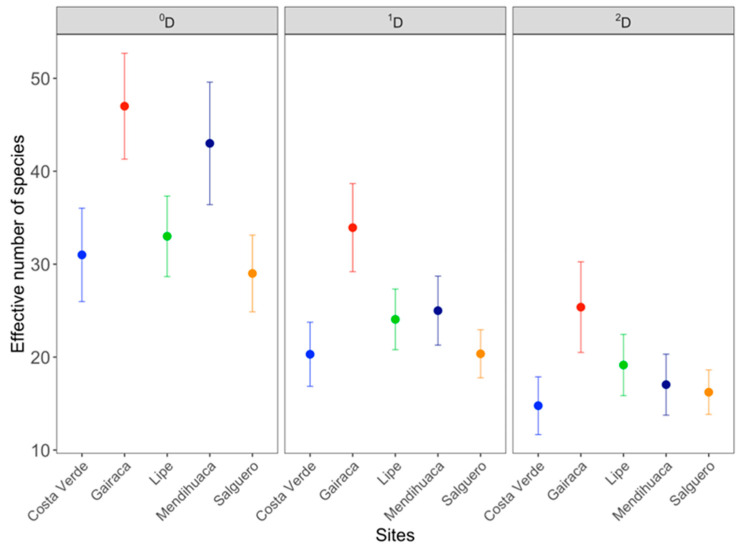
Alpha plant diversity of five beaches from the northern Colombian Caribbean. The following were evaluated: ^0^*D*, species richness; ^1^*D*, number of common species; and ^2^*D*, dominant species in the community. Error bars correspond to the 95% confidence interval.

Analyses performed with 95% confidence intervals (CI) showed significant differences for the diversity orders (^0^*D,* ^1^*D*, ^2^*D)* between the beaches studied. Species richness (^0^*D*) was similar between Gairaca and Mendihuaca and was significantly higher at these sites than at the other three (Figure 4). For the ^1^*D* order, Gairaca showed significantly higher values than the others, indicating greater evenness. Finally, for the ^2^*D* order, the true diversity values gradually decreased from the most preserved site, Gairaca (with the lowest dominance), to the least, Costa Verde (Figure 4).

The total Jaccard dissimilarity (b_jac_) among sites was generally high (>0.80) (Table 1), indicating marked heterogeneity in species composition. The highest turnover-driven dissimilarity values were observed between Mendihuaca and Lipe, as well as between Mendihuaca and Gairaca (the most preserved site) and between Mendihuaca and Costa Verde (the most disturbed site), (Table 1). However, Mendihuaca exhibited the highest dissimilarity values compared with the other sites, confirming it as the most distinct beach in terms of biological composition.

In contrast to species turnover, nestedness (βjn) showed low values (<0.05), indicating that variation in species richness contributed little to the observed differences. Slight nestedness contributions were recorded only between Gairaca and Lipe, and between Gairaca and Salguero, albeit at minimal levels. Although the nestedness-related dissimilarity observed between Gairaca and Lipe showed the highest value, it remained within a low nestedness range, suggesting that part of Lipe’s plant community constitutes a weak subset of that of Gairaca. As both sites are highly conserved, this difference does not reflect degradation but may instead be driven by subtle variations in species richness associated with environmental microheterogeneity and local geomorphological differences. (Table 1).

### 2.5. Variation in Community Structure

**Figure 5:**
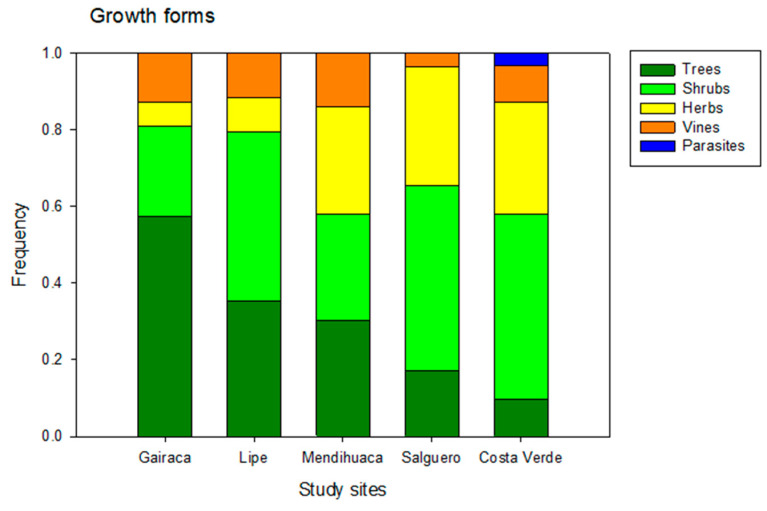
Frequency of different plant growth forms in five beaches along the Colombian Caribbean, exposed to increasing anthropogenic disturbances. Gairaca was the least disturbed, and Costa Verde was the most.

Trees and shrubs were more abundant in the best-preserved locations, while grasses were predominant in the most disturbed ones (Figure 5). Thus, in Gairaca, located within a national park, trees such as *Morisonia odoratissima* and *M. tenuisiliqua* (Capparaceae), *Platymiscium pinnatum* and *Senegalia tamarindifolia* (Fabaceae), and *Ximenia americana* (Olacaceae) were abundant. Other trees were found on Lipe beach, *Pithecellobium dulce* (Fabaceae), *Astronium graveolens* (Anacardiaceae), *Bonellia frutescens* (Primulaceae), *Tecoma stans* (Bignoniaceae), and *Guaiacum officinale*. In turn, perennial herbs (*Sesuvium portulacastrum*—Aizoaceae and *Sporobolus virginicus*—Poaceae) were abundant on the most disturbed beaches, Salguero and Costa Verde.

The highest relative frequency of tree plants, occurring in Gairaca (60%), indicates a more mature and well-preserved vegetation formation compared to the other beaches. Lipe (35%) and Mendihuaca (30%) had lower proportions of trees, reflecting a mixture of other life forms, such as shrubs or herbaceous plants (Figure 5). Finally, Salguero (18%) and Costa Verde (10%) have very low tree cover, which could indicate open vegetation dominated by herbaceous plants and shrubs in degraded areas. Costa Verde was the only location where parasitic plants were observed (Figure 5).

**Figure 6:**
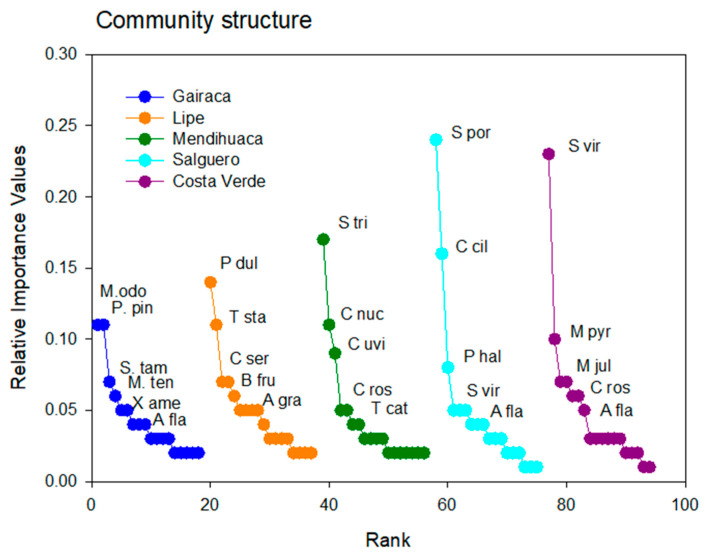
Relative Importance Values of plant species growing on the beach and coastal dunes in five beaches along the Colombian Caribbean, exposed to increasing anthropogenic disturbances, with Gairaca being the least disturbed and Costa Verde the most. The most important species are indicated in each beach through abbreviated nomenclature: M. odo (*Morisonia odoratissima*), P. pin (*Platymiscium pinnatum*), S. tam (*Senegalia tamarindifolia*), M. ten (*Morisonia tenuisiliqua*) E. hon (*Erythroxylum hondense*), A. fla (*Althernantera flavescens*), P. dul (*Pithecellobium dulce*), T sta (*Tecoma stans*), A. grav (*Astronium graveolens*) C. ser (*Chamaecrista serpens*), B. fru (*Bonellia frutescens*), S. tri (*Sphagneticola trilobata*), C. nuc (*Cocos nucifera*), C. uvi (*Coccoloba uvifera*), C. ros (*Canavalia rosea*), S. por (*Sesuvium portulacastrum*), C. cil (*Cenchrus ciliaris*), S. vir (*Sporobolus virginicus*), P. hal (*Portulaca halimoides*), M. pyr (*Melochia pyramidata*). N. Jul (*Neltuma juliflora*), I. pes (*Ipomoea pes-caprae*).

The Rank–Importance curves varied between sites (Figure 6), and the important species changed notably, with only a few being widely represented. The curve for Gairaca is indicative of a more equitative community with trees such as *Morisonia odoratissima* and *Platymiscium pinnatum* being the dominant species. In contrast, herbs such as *Sesuvium portulacastrum* and *Sporobolus virginicus* were clearly dominant in Salguero and Costa Verde (Figure 5), and the plant communities here were less equitative and dominated by one species.

Interestingly, the dominant species in one location were relatively scarce in other locations. For instance, *Sporobolus viginicus,* the dominant species in Costa Verde, was very scarce on other sites. The same occurred for *Sesuvium portulacastrum*, the dominant species in Salguero and very scarce elsewhere. Similar trends were observed in the other sites, where the dominant species were absent (Figure 6).

### 2.6. The Role of Environmental Variables in Plant Communities

**Figure 7:**
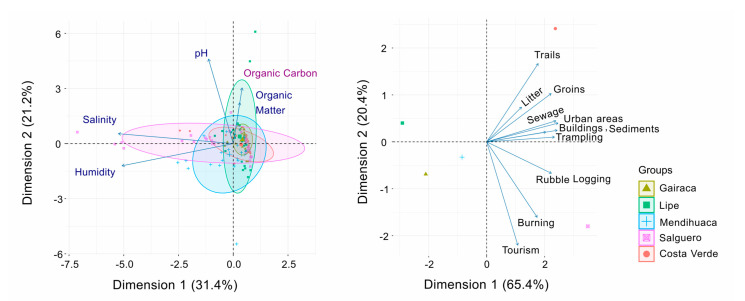
Canonical correspondence analyses showing how natural (**left**) and anthropogenic (**right**) variables affect community composition of plant species growing on the beach and coastal dunes in five locations along the Colombian Caribbean, exposed to increasing anthropogenic disturbances, with Gairaca being the least disturbed and Costa Verde the most.

The Canonical Correspondence Analysis shows the influence of the gradient of environmental variables—salinity, humidity, pH, organic matter (OM), and organic carbon (OC)—on the distribution of plant species among sites. Axis 1 and 2 explained 31.4% and 21.2%, respectively, accumulating 52.5% of the total variance of the environmental variables (Figure 7). The variables pH, OM, and CO exhibited similar vector lengths. Salguero was correlated with pH, salinity and humidity. However, the floristic groups formed were not clearly distinguishable, indicating that, in general, the correlation between plant species and natural environmental sites was similar across sites.

In turn, the Canonical Correspondence analysis performed with anthropogenic variables clearly showed how they were most relevant in the most disturbed sites: Salguero and Costa Verde (Figure 7). Axis 1 and 2 explained 65.4% and 20.4%, respectively, accounting for 85.8% of the total variance in the anthropogenic variables. In this case, the anthropogenic variables clearly showed differences between sites.

## 3. Discussion

This study shows that natural and anthropogenic factors varied between the study sites, which were correlated with the composition and structure of the plant communities from the beach and coastal dunes along the Colombian Caribbean coast. Human disturbances (urbanized areas, construction, burning, debris, trampling, logging, tourism, groins, sewage, roads, garbage, and sediment extraction) were particularly relevant. The sites less affected by human activities showed higher plant cover and species diversity, a higher percentage of trees and vines, and reduced dominance by any single species. In turn, the opposite occurred in the disturbed sites, characterized by lower plant cover and diversity, abundant shrubs and parasitic plants, and the dominance of a few herbaceous species. Management alternatives should account for environmental factors affecting vegetation to improve the conservation of plant biodiversity in the coastal dunes along Colombia’s Caribbean coast.

### 3.1. Environmental Factors: Natural and Anthropogenic

Natural characteristics and disturbances may limit species diversity and abundance, leaving only those with the necessary adaptations to survive the site’s specific conditions [53,54]. Among the observed natural factors that may be modulating biodiversity were geomorphological dune attributes (dune height and slope) and beach characteristics (beach width and the area covered by vegetation [55]). The high plant cover of sandy dunes observed in Gairaca, Lipe, and Mendihuaca suggests that they are stabilized and help retain sand, preventing wind erosion and promoting the accumulation of more sediments.

Considering the environmental ocean-land gradient, saltwater flooding (salinity) in the zone closest to the sea limits plant growth, and only those adapted to saline environments remain. For instance, areas exposed to regular flooding by ocean waves in Mendihuaca promoted the dominance of species such as *S. trolibata*, which is tolerant to salinity. Similar results were observed by Bernal et al. [56], who highlighted the dominance of herbaceous plants such as *S. trilobata* in association with gravel and sand, low slopes, and seawater flooding. *S. trilobata* grows in coastal dunes and structureless soils, with high salinity, temperatures, and direct exposure to solar radiation [57,58,59].

High anthropogenic impacts were observed to coincide with those reported in previous studies, such as those by Pereira et al. [36,55,60]. These authors mention urbanization and garbage problems [61], the presence of invasive plants such as *Calotropis procera* and *Cryptostegia madagascariensis* [62], sand extraction, and increased human traffic [55] at disturbed sites.

At the most disturbed sites, Salguero and Costa Verde, burning, the construction of housing and tourism infrastructure, and the installation of groins have resulted in habitat loss and alteration of the natural landscape [63,64]. These beaches are also affected by trampling, roads, and excessive logging, which negatively affect germination, reproduction, survival, species richness, cover, and diversity [63,65]. In general, human disturbances promoted the invasibility of coastal vegetation by facilitating the colonization of new propagules of exotic plant species, resulting in the loss of native species [62,66,67] such as *Calotropis procera* and *Tribulus cistoides*. Additionally, *Cenchrus ciliaris*, *Chloris barbata*, *Tephrosia purpuria*, *Cocos nucifera*, and *Azadirachta indica* are also negatively affected by human disturbances.

Vegetation structure also varied between sites. Better-preserved beaches support more diverse plant communities dominated by woody species, which increase surface roughness and enhance sediment retention, contributing to more mature and stable dune systems [68,69,70]. In contrast, disturbed beaches exhibit simplified communities dominated by disturbance-tolerant herbaceous species, with a reduced capacity for dune stabilization under intense urbanization [49,71,72]. The high intensity of anthropogenic factors observed at less-conserved sites disrupts biogeomorphological feedbacks, reducing the provision of ecosystem services and compromising the long-term stability and sedimentary dynamics of coastal dune systems [16,73,74].

### 3.2. Floristic Composition and Community Structure

A total of 133 species, included in 112 genera and 53 families, were identified on the beach coastal dunes of the northern Colombian Caribbean. These results improve previous findings in the area, which have reported a maximum of 70 species and 38 families [75]. The dominance of the Fabaceae family (26 species) is consistent with previous reports [36,76].

The dominant families align with findings from other beaches and coastal dunes. For example, studies in the Venezuelan Caribbean highlight the diversification of families present in beaches and dunes: Poaceae (15), Cyperaceae (11), Fabaceae (8), Euphorbiaceae (5), Boraginaceae (5), Malvaceae (5), and Asteraceae (4), among others, for a total of 97 species with 73 genera included in 34 families [26]. A high biodiversity from Mexican coastal dunes [77] reports 153 families, included in 897 genera, and 2075 species. Again, Fabaceae and Poaceae were the most abundant families with the greatest number of species. Plant diversity in the Brazilian Catinga is lower than in the aforementioned studies, with 86 species across 37 families. Once more, the Fabaceae family (24 species) was considered the most important due to its richness [78]. In all cases, fewer plant species is typical of beach and coastal dune environments. The plant cover of stabilized dunes contains species from surrounding vegetation types, including tropical forests, scrublands, mangroves, and freshwater wetlands [77], thus contributing to the biodiversity of the study sites.

Two species are worth mentioning. Among the Poaceae, the most common dune-forming species was *Sporobolus virginicus*, dominant in Salguero and Costa Verde, the two the most disturbed beaches. Espejel et al. [77] indicate that *Sporobolus virginicus* is the most common grass on beaches and embryo dunes, occupying large areas. This grass tolerates salinity and sand movement [14], which explains its occurrence along the transects, especially close to the ocean. In addition to *Sporobolus virginicus*, clumps of *Cenchrus ciliaris*, a species native to Colombia, are frequently found; in short, these grasses are more common in disturbed environments.

In addition to *S. virginicus*, *Morisonia* (Capparaceae) was the most representative genus with seven species. This genus was recorded on preserved beaches such as Gairaca and Lipe, with 6 and 2 species, respectively, indicating that Morisonia odoratissima is common on these beaches and absent on the remaining beaches. The abundance of trees and shrubs at the preserved sites (Gairaca and Lipe) aligns with previous studies [78] on beaches of San Bernardo Island, where more than half of the sampled species were woody. On the other hand, Rangel [36] and Gomez et al. [14] identified herbaceous growth habit as the most important in their studies, consistent with results observed on beaches with high anthropogenic disturbance.

In line with the results on species richness, biodiversity was highest in the preserved sites (Gairaca and Lipe) compared to those with more intense anthropogenic activity (Salguero and Costa Verde). This finding aligns with the study by Rangel Buitrago [36], which showed a reduced biodiversity due to anthropogenic impact. It is interesting to note that Mendihuaca, a moderately disturbed site due to anthropogenic factors, exhibited high diversity. This finding was probably the result of a combination of species typical of preserved sites coexisting with species from moderately perturbed locations, which follows Connell’s [79] intermediate disturbance hypothesis and coincides with the study by Pinna et al. [80]. Logging was a key anthropogenic driver that promoted opportunistic species, while the introduction of edible and ornamental plants increased observed species richness (^0^D) and led to high species dominance (^2^D) due to disturbance.

### 3.3. Management Alternatives

Effective management of the beach and coastal dunes along Magdalena’s coastline requires several approaches. Locally, due to intensive tourism activities, it is necessary to estimate and monitor the carrying capacity of each beach [81,82,83]. Also, regulating trampling through boardwalks, designated access points, and restricted areas is necessary to recover natural vegetation [84].

At a broader territorial scale, several regulations govern beach setbacks for construction on consolidated land, which may vary based on beach dynamics. Nonetheless, along the coastline of Magdalena, many buildings are close to the high tide line. The disruption of coastal dynamics exacerbates erosion, thereby generating both ecological and economic risks. In the Colombian Caribbean, coastal erosion issues affecting these constructions and settlements in high-risk areas have been documented, with mitigation relying primarily on hard-engineering solutions. These interventions often have severe impacts, underscoring the need for preventive approaches and soft-engineering alternatives [5,61,85].

Establishing clear state policies is essential to delineate and protect natural protected areas within these ecosystems. Such policies could be aligned with international certifications such as the Blue Flag and hotel sustainability labels. Furthermore, in degraded areas, ecological restoration programs with native species, invasive species control, and ecotourism promotion are necessary. Regulatory strategies for resource extraction and effective waste management are also required to safeguard the integrity of coastal ecosystems, as waste accumulation undermines scenic and recreational value and threatens the regeneration and stability of dune systems [61]. Finally, awareness-raising initiatives that foster the social appropriation of these ecosystems, along with participatory environmental monitoring, are necessary to alleviate pressures and ensure the maintenance of the ecosystem services provided by dunes.

## 4. Materials and Methods

### 4.1. Study Sites

**Figure 8:**
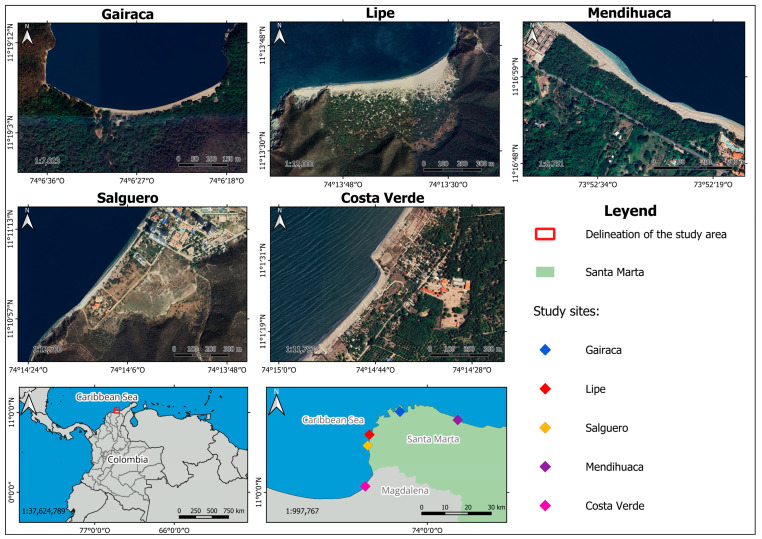
Location of the study sites in the northern Colombian Caribbean. Satellite images from Google Earth.

The study area included coastal dunes from five beaches: Gairaca (Tayrona National Natural Park), Lipe, Mendihuaca, Salguero, and Costa Verde in the department of Magdalena, in the Colombian Caribbean (Figure 8, Table 2). Five sites were selected along the coastline with different degrees of anthropogenic and natural stressors: (a) Gairaca (moderately visited beach within a National Natural Park; (b) Lipe (beach exposed to natural events and protected by its geomorphological conditions; (c) Mendihuaca (beach moderately visited by tourists; (d) Salguero (highly visited by tourists) and (e) Costa Verde (with unplanned urban and tourism expansion and crops).

The climate in the coastal zone is warm and dry, with an average annual temperature of 28 °C, a minimum of 25 °C, and a maximum of 34 °C. The mean annual rainfall ranges from 362 to 500 mm [86]. The climate is determined by the influence of the Sierra Nevada de Santa Marta (5900 m), the trade winds, and ocean currents [87,88]. The precipitation regime is defined by the Intertropical Convergence Zone (ITCZ), resulting in a bimodal pattern with two rainy periods: April to June and August to November, alternating with two dry seasons: December to March and June to August. However, below 200 m above sea level, the system is monomodal: the north face experiences a tropical climate with precipitation from July to November and a single well-marked dry period from December to June. In contrast, the coastal area of the northwestern face is characterized by four distinctive periods: major dry (December–April), minor rainy (May–June), minor dry (July–August), and major rainy (September–November) [89]. Consequently, the north face is the wettest (Mendihuaca and Gairaca), the eastern face is the driest (towards the interior of the continent), and the northwestern face shows an intermediate condition (Lipe, Salguero, and Costa Verde beaches).

### 4.2. Environmental Variables: Natural and Anthropogenic

The environmental variables used in this study included topography (dune height, slope), beach width, vegetation width, and plant cover. Dune topography was measured starting at a point of known height (x = 0 and y = 0). Then, vertical distances were measured using a laboratory-made inclinometer, following a previously described methodology [27]. The instrument allowed measurement of changes in topography between two points spaced 0.5 or 1 m apart, depending on the topography.

At each site, three profiles were measured from the dunes toward the ocean, and in each, we estimated the maximum height (m) of the frontal dune as the vertical distance from sea level to the dune crest. Using the x, y coordinates, the slope of the dune along each profile was calculated as the slope of the straight line (Equation (1)) and then expressed as a percent change (Equation (1)).

(1) $$m=\frac{y_{2}-y_{1}}{x_{2}-x_{1}}$$

where x_1_ and y_1_ correspond to the coordinates of the first point of the segment (initial distance and height), and x_2_ and y_2_ represent the coordinates of the second point (final distance and height) of the segment, and thus, the inclination was calculated.

Other environmental variables measured in the field included the width of the beach (measured with a measuring tape), from the highest water mark to the beginning of the foredune. Tides in Colombia are very narrow with no significant changes throughout the day. We also measured the width of the vegetation-covered area and the mean plant cover per plot.

In addition, sedimentological variables were assessed. Three sediment samples were collected from each plot, using three subsamples taken from the top 15 cm of soil, for a total of 400 g [15,90]. In each sample, we measured pH of the sand, moisture, salinity, organic matter, and carbon [91].

Twelve anthropogenic variables were used: (1) tourism, (2) logging, (3) trampling, (4) burning, (5) sediment extraction, (6) garbage, (7) debris, (8) constructions, (9) roads, (10) sewage, (11) spurs, and (12) urban areas. We used ordinal values for each variable, which varied from 1 to 4. The ordinal scale of presence/intensity was used as follows: 1 indicates the absence of the variable at the site, while values 2–4 indicate the presence of the anthropogenic effect, with higher values indicating greater intensity. The dataset was analyzed in RStudio using the Mirt package, applying a modified item–person map (also known as an un-Wright map) to visualize the relative intensity and distribution of anthropogenic factors across sites [92].

### 4.3. Vegetation Sampling

At each site, transects of different lengths were carried out perpendicular to the coastline until there was a change in the morphology of the terrain and vegetation. The number of transects varied according to the sampling saturation. Thus, we had three transects in Gairaca and Costa Verde; four in Lipe, and six in Mendihuaca and Salguero, giving a total of 22 sampled transects. Along each transect, nine 2 × 2 m plots were set, interspersed on the right and left, for a total of 27 to 54 plots per site. The sampling numbering was organized with the plot closest to the sea designated as 1 and plot 9 as the most distant [21,90]. Plant cover per species was visually estimated in each plot.

Plant samples were collected from each plot, pressed, and herbarized [93]. The collection of biological material was deposited at the Center for Biological Collections (CBUMAG) of the University of Magdalena. The floristic composition was determined using specialized morphological taxonomic keys for the different groups. The identifications were corroborated using reference virtual herbaria such as the Colombian National Herbarium (COL) (http://www.biovirtual.unal.edu.co, accessed on 12 December 2025), the New York Botanical Garden (NY), and Tropicos (http://www.tropicos.org, accessed on 12 December 2025), along with consultation with specialist herbaria and visits to the COL herbarium to clarify difficult identifications. The scientific names and growth habits were updated with POWO (https://powo.science.kew.org/, accessed on 12 December 2025) and The WFO Plant List (https://wfoplantlist.org/, accessed on 12 December 2025).

### 4.4. Data Analyses

First, alpha diversity was determined for each site using the effective number of species [94], following the Hill series [95] and the qD notation (Equation (2)) in RStudio.

(2)  Dq=∑i=lSpiq1(1−q)

where ^q^D is the community diversity according to the selected index, ^q^ exponent and superscript is the “order of diversity”, *pi* is the relative abundance (proportional abundance) of each species, and *S* is the number of species. In this case, ^q^ = 0 (^0^*D*) represents true species richness, *q* =1 (^1^*D*) represents common species (Shannon diversity), and *^q^* = 2 (^2^*D*) represents dominant species (Simpson diversity) [94]. Comparisons of diversity values were performed using coefficient intervals (CIs) overlapping at 95% [96]. Analyses were performed with the iNEXT library [97] in R Studio (R version 3.6.1 “Action of the Toes”) [98].

Second, beta diversity was calculated based on the dissimilarity between sites by computing Jaccard’s dissimilarity index (βjac) (Equation (3)). The two partitioning components were considered: species turnover (b_jtu_) and species nestedness (b_jne_) [99] to better interpret biodiversity patterns [99,100].

(3) $$\beta_{jac}=\frac{b+c}{a+b+c}$$

where a is the number of species shared across both sites, b is the number of species found at the first site but not at the second, and c is the number of species found at the second site but not at the first [100]. The index ranges from 0 (zero dissimilarity) to 1 (complete dissimilarity) [100]. Analyses were performed using the betapart package [100] in R Studio version 4.4.1.

Third, the species importance value index (IVI) was estimated per site, *IVI* = [(*Fr* + *Cr*)/2], where the relative frequency (*Fr*) is defined as the number of plots where the species appears, and the relative cover (*Cr*) is the plant cover per species divided by the total plant cover. The cover of each species, estimated as a percentage within each plot, was converted into square meters for the IVI calculation. The relative cover was considered as the total cover of the species in the plots where it was present, divided by the total cover of all species in the plots [71]. The IVI obtained a maximum value of 1 to facilitate interpretation [71,101].

Finally, a Canonical Correspondence Analysis was performed to examine the correlations between environmental variables (natural and anthropogenic) and plant communities in terms of species composition. The Vegan R library was used to conduct this analysis [102].

## 5. Conclusions

Natural and anthropogenic factors varied across the study sites and were correlated with the composition and structure of plant communities along the beach and coastal dunes of the Colombian Caribbean coast. Specifically, human activities such as urban expansion, infrastructure development, trampling, logging, burning, road construction, and tourism represented the main drivers affecting the coastal dune vegetation. In Salguero and Costa Verde, these pressures have led to a marked reduction in vegetation cover, species richness, and diversity. The high-intensity anthropogenic pressures require implementing integrated coastal management strategies to reverse degradation and promote ecological recovery. This should include reducing and controlling anthropogenic pressures, promoting active ecological restoration of dune vegetation and geomorphology at these sites, and monitoring the effectiveness of the measures implemented. In contrast, sites such as Gairaca and Lipe exhibit better conservation status, characterized by lower levels of human disturbance, greater vegetation cover, and higher abundance of arboreal species. Overall, the best-preserved sites are associated with more stable geomorphological conditions, particularly higher dune heights and greater vegetation cover.

## Figures and Tables

**Table 1:** Jaccard’s dissimilarity Index and its Turnover and Nestedness components between five beach–coastal dune systems along the Colombian Caribbean coast. Values closest to 1 show maximum dissimilarity between sites (the sites do not share any species). Bold letters highlight the highest values. Sites are organized from least (Gairaca) to most disturbed (Salguero).

Total Dissimilarity (β_jac_)					
	Gairaca	Lipe	Mendihuaca	Costa Verde	Salguero
Gairaca	0				
Lipe	0.805	0			
Mendihuaca	**0.941**	**0.986**	0		
Costa Verde	0.916	0.915	0.895	0	
Salguero	0.898	0.680	**0.985**	0.846	0
Turnover component (β_jtu_)					
	Gairaca	Lipe	Mendihuaca	Costa Verde	Salguero
Gairaca	0				
Lipe	0.754	0			
Mendihuaca	**0.938**	**0.984**	0		
Costa Verde	0.892	0.912	0.872	0	
Salguero	0.862	0.651	**0.982**	0.840	0
Nestedness component (β_jne_)					
	Gairaca	Lipe	Mendihuaca	Costa Verde	Salguero
Gairaca	0				
Lipe	**0.051**	0			
Mendihuaca	0.002	0.002	0		
Costa Verde	0.023	0.002	0.022	0	
Salguero	**0.035**	**0.029**	0.003	0.006	0

**Table 2:** Characteristics of the study sites located along the Northern Colombian Caribbean coast. Sites are organized from least to most disturbed.

Study Site	Dimensions	Location	Tourism	Environmental Attributes	Anthropogenic Activities	Sand
Gairaca	547 × 147 m	Located in a protected Natural Area	Medium intensity tourism	Located between two hills. Moderately accessible	Some houses, some food provision	pH = 7.1 (0.9); Sal = 0.1 (0.1); Cond = 357 (191); Hum = 1.6 (1.9); OM = 1.7 (0.9)
Lipe	627.17 × 394.38 m	Close to the city of Santa Marta and a river	Low tourism.	Limited accessibility through paths and by boat	Some paths.	pH = 6.9 (1.1); Sal = 0.1 (0.3); Cond = 215 (232); Hum = 3.1 (2.3); OM = 0.9 (0.8)
Mendihuaca	562.28 × 120.5 m	Close to the Mendihuaca River	Moderate-density houses, hotels, and restaurants	Easily accessible	Local inhabitants and tourists visit the beach.	pH = 6.5 (0.6); Sal = 0.2 (0.5); Cond = 478 (975); Hum = 6.9 (6.9); OM = 1.4 (0.9)
Salguero	547.67 × 584.94 m	Close to the Gaira River.	High-density hotels	Easily accessible	High-intensity tourism.	pH = 6.9 (0.8); Sal = 0.1 (4); Cond = 8141 (18,553); Hum = 5.4 (6.7); OM = 1.5 (1.1)
Costa Verde	862.85 × 471.82 m	Close to Costa Verde mangroves and the Córdoba river.	Unplanned urban and tourism expansion.	Easily accessible	Growing tourism, crops.	pH =6.8 (0.8); Sal = 0.2 (0.6); Cond = 363 (746); Hum = 2.2 (4.2); OM = 1.8 (0.8)

## Data Availability

The collected plant material was deposited in the Scientific Collections Center of the Universidad del Magdalena (CBUMAG). Each specimen was taxonomically identified and labeled, with detailed collection data including georeferencing, date, and habitat description. *Guaiacum officinale*, an endangered species, was photographically documented. All specimens are available for public consultation. The collection was conducted within the framework of the project “Functional traits of vegetation and their relationship with environmental and geomorphological variables.” Specimens are registered in the Biodiversity Information System (SIB). The data used in this article can be made available upon request to the authors.

## Appendix A

**Table A1:** Species identified on the beach coastal dunes of the northern Colombian Caribbean. Study sites are listed from most to least preserved: G = Gairaca, L = Lipe, M = Mendihuaca, S = Salguero, and CV = Costa Verde.

Family	Species	G	L	M	S	CV
Fabaceae	*Alysicarpus vaginalis* (L.) DC.					x
	*Canavalia rosea* (Sw.) DC.	x		x		x
	*Chamaecrista serpens* (L.)		x		x	
	*Coulteria mollis* Kunth	x				
	*Coursetia caribaea* (Jacq.) Lavin	x				
	*Dalbergia brownei* (Jacq.) Schinz			x		
	*Desmodium* sp.			x		x
	*Gliricidia sepium* (Jacq.) Kunth			x		
	*Guilandina bonduc* L.			x		
	*Leucaena trichodes* (Jacq.) Benth.	x				
	*Libidibia punctata* (Willd.) Britton	x				
	*Mimosa arenosa* (Willd.) Poir.	x	x			
	*Muellera sanctae-marthae* (Pittier) M.J.Silva & A.M.G. Azevedo	x				
	*Neltuma juliflora* (Sw.) Raf.	x	x		x	x
	*Neptunia plena* (L.) Benth.					x
	*Pithecellobium dulce* (Roxb.) Benth.	x	x		x	x
	*Platymiscium pinnatum* (Jacq.) Dugand	x	x			
	*Pterocarpus officinalis* Jacq.			x		
	*Senegalia tamarindifolia* (L.) Britton & Rose	x				
	*Senna atomaria* (L.) H.S.Irwin & Barneby	x				
	*Sesbania sesban* (L.) Merr.					x
	*Stylosanthes humilis* Kunth					x
	*Tephrosia cinerea* (L.) Pers.	x	x			
	*Tephrosia purpurea* (L.) Pers.				x	x
	*Vachellia tortuosa* (L.) Seigler & Ebinger		x		x	
	*Vigna marina* (Burm.) Merr.			x		
Poaceae	*Cenchrus ciliaris* L.		x		x	
	*Cenchrus pilosus* Kunth					x
	*Chloris barbata* Sw.				x	
	*Chloris virgata* Sw.					x
	*Coix lacryma-jobi* L.			x		
	*Cynodon dactylon* (L.) Pers.				x	
	*Eleusine tristachya* (Lam.) Lam.					x
	*Eragrostis* sp.			x		
	*Panicum* sp.	x		x		
	*Sporobolus virginicus* (L.) Kunth	x			x	x
Malvaceae	*Corchorus aestuans* L.			x		x
	*Guazuma ulmifolia* Lam.	x		x		
	*Malvastrum coromandelianum* (L.) Garcke		x			
	*Melochia crenata* Vahl		x			
	*Melochia pyramidata* L.					x
	*Melochia tomentosa* L.	x	x		x	
	*Pseudabutilon umbellatum* (L.) Fryxell	x				
	*Waltheria indica* L.					x
Euphorbiaceae	*Cnidoscolus urens* (L.) Janti		x		x	
	*Croton ovalifolius* Vahl		x			
	*Euphorbia hirta* L.			x		
	*Euphorbia hyssopifolia* L.			x		
	*Jatropha gossypiifolia* L.				x	
	*Manihot carthagenensis* (Jacq.) Müll.Arg.	x				
	*Sapium glandulosum* (L.) Morong			x		
Capparaceae	*Morisonia americana* L.	x				
	*Morisonia indica* (L.) ined.	x				
	*Morisonia linearis* (Jacq.) Christenh. & Byng		x			
	*Morisonia odoratissima* (Jacq.) Christenh. & Byng	x	x			
	*Morisonia pachaca* (Kunth) Christenh. & Byng	x				
	*Morisonia tenuisiliqua* (Jacq.) Christenh. & Byng	x				
	*Morisonia verrucosa* (Jacq.) Christenh. & Byng	x				
Cactaceae	*Acanthocereus tetragonus* (L.) Hummelinck		x			
	*Leuenbergeria guamacho* (F.A.C.Weber) Lodé	x	x		x	
	*Opuntia caracassana* Salm-Dyck		x		x	
	*Pilosocereus lanuginosus* (L.) Byles & G.D.Rowley		x		x	
	*Stenocereus griseus* (Haw.) Buxb.		x		x	
Polygonaceae	*Coccolaba acuminata* Kunth				x	
	*Coccoloba obtusifolia* Jacq.	x				
	*Coccoloba uvifera* (L.) L.			x		x
	*Enneatypus ramiflorus* (Jacq.) Roberty & Vautier.		x			
Rubiaceae	*Morinda royoc* L.			x		
	*Oldenlandia corymbosa* L.					x
	*Spermacoce exilis* (L.O.Williams) C.D.Adams ex			x		
	*Spermacoce latifolia* Aubl.			x		
Bignoniaceae	*Crescentia cujete* L.	x				
	*Handroanthus billbergii* (Bureau & K.Schum.) S.O.Grose	x				
	*Tecoma stans* (L.) Juss. ex Kunth		x			
Convolvulaceae	*Ipomoea incarnata (Vahl) Choisy*	*x*				
	*Ipomoea carnea Jacq.*	*x*				
	*Ipomoea pes-caprae (L.) R.Br.*	*x*		*x*		*x*
Cyperaceae	*Cyperus* sp1					x
	*Cyperus* sp2			x		x
	*Fimbristylis* sp					x
Apocynaceae	*Calotropis procera* (Aiton) W.T.Aiton		x		x	x
	*Ibatia maritima* (Jacq.) Decne.	x	x	x		
Asteraceae	*Sphagneticola trilobata* (L.) Pruski			x		
	*Trixis inula* Crantz	x				
Boraginaceae	*Cordia dentata* Poir.				x	
	*Varronia bullata* L.				x	
Burseraceae	*Bursera graveolens* (Kunth) Triana & Planch.	x				
	*Bursera simaouruba* (L.) Sarg.	x				
Erythroxylaceae	*Erythroxylum carthagenense* Jacq.	x				
	*Erythroxylum hondense* Kunth	x				
Moraceae	*Ficus insipida* Willd.			x		
	*Maclura tinctoria* (L.) D.Don ex G.Don			x		
Portulacaceae	*Portulaca halimoides* L.				x	
	*Portulaca pilosa* L.		x			
Verbenaceae	*Lantana camara* L.			x		
	*Phyla nodiflora* (L.) Greene					x
Zygophyllaceae	*Guaiacum officinale* L.		x			
	*Tribulus cistoides* L.		x		x	x
Acanthaceae	*Ruellia blechum* L.	x				
Aizoaceae	*Sesuvium portulacastrum* (L.) L.				x	x
Amaranthaceae	*Alternanthera flavescens* Kunth	x	x		x	x
Amaryllidaceae	*Hymenocallis littoralis* (Jacq.) Salisb.			x		
Anacardiacee	*Astronium graveolens* Jacq.	x	x			
Arecaceae	*Cocos nucifera* L.			x		x
Chrysobalanaceae	*Parinari pachyphylla* Salm-Dyck			x		
Combretaceae	*Terminalia catappa* L.			x		
Commelinaceae	*Commelina* sp.			x		
Cucurbitaceae	*Momordica charantia* L.			x		
Hernandiaceae	*Gyrocarpus americanus* Jacq.	x				
Linderniaceae	*Lindernia dubia* (L.) Pennell			x		
Loganiaceae	*Spigelia anthelmia* L.			x		
Loranthaceae	*Passovia pedunculata* (Jacq.) Kuijt					x
Meliaceae	*Azadirachta indica* A.Juss.					x
Molluginaceae	*Mollugo verticillata* L.			x	x	
Monimiaceae	*Mollinedia ovata* Ruiz & Pav.	x				
Muntingiaceae	*Muntingia calabura* L.			x		
Musaceae	*Musa acuminata* Colla			x		
Myrtaceae	*Psidium guajava* L.			x		
Nyctaginaceae	*Commicarpus scandens* (L.) Standl.		x			
Olacaceae	*Ximenia americana* L.	x	x			
Passifloraceae	*Passiflora foetida* L.					x
Petiveriaceae	*Petiveria alliacea* L.	x				
Phyllanthaceae	*Phyllanthus urinaria* L.			x		
Piperaceae	*Piper bredemeyeri* J.Jacq.			x		
Primulaceae	*Bonellia frutescens* (Mill.) B.Ståhl & Källersjö		x		x	
Sapindaceae	*Cardiospermum halicacabum* L.	x	x			
Sapotaceae	*Sideroxylon obtusifolium* (Roem. & Schult.) T.D.Penn.	x			x	
Schizaeaceae	*Lygodium venustum* Sw.			x		
Solanaceae	*Solanum incanum* L.			x		
Talinaceae	*Talinum fruticosum* (L.) Juss.				x	
Tetrachondraceae	*Polypremum procumbens* L.			x		
Urticaceae	*Cecropia peltata* L.			x		
Vitaceae	*Cissus trifoliata* (L.) L.		x			
